# The genome sequence of the Black Rustic, *Aporophyla nigra* (Haworth, 1809)

**DOI:** 10.12688/wellcomeopenres.19339.1

**Published:** 2023-04-17

**Authors:** David Lees, Natural History Museum Genome Acquisition Lab, Darwin Tree of Life Barcoding collective, Wellcome Sanger Institute Tree of Life programme, Wellcome Sanger Institute Scientific Operations: DNA Pipelines collective, Tree of Life Core Informatics collective, Darwin Tree of Life Consortium

**Affiliations:** 1 Natural History Museum, London, England, UK

**Keywords:** Aporophyla nigra, Black Rustic, genome sequence, chromosomal, Lepidoptera

## Abstract

We present a genome assembly from an individual male *Aporophyla nigra* (the Black Rustic; Arthropoda, Insecta, Lepidoptera, Noctuidae). The genome sequence is 736.9 megabases in span. Most of the assembly is scaffolded into 31 chromosomal pseudomolecules, including the Z sex chromosome. The mitochondrial genome has also been assembled and is 16.0 kilobases in length.

## Species taxonomy

Eukaryota; Metazoa; Ecdysozoa; Arthropoda; Hexapoda; Insecta; Pterygota; Neoptera; Endopterygota; Lepidoptera; Glossata; Ditrysia; Noctuoidea; Noctuidae; Xyleninae; *Aporophyla*; *Aporophyla nigra* (Haworth, 1809) (NCBI:txid988065).

## Background

The Black Rustic, *Aporophyla nigra*, is a medium sized noctuid moth, its upper wings almost jet black and rather glossy, the reniform stigma edged with white. It emerges in the temperate Autumn, flying in September and October in most of the UK, or August in Scotland, overwintering as a larva ( Waring *et al.*, 2017).

*A. nigra* is found in a wide variety of open habitats including those in woodlands. The larva is polyphagous on a wide range of herbaceous plants and shrubs. Adults seek nectar such as that of Ivy ( *Hedera helix* L.) at night, and also feed on ripe blackberries.

The Black Rustic is generally common and widespread in the western Palaearctic only, from western Scandinavia to the northern shores of the Mediterranean and Black Sea, and Middle East, while there are relatively few records for eastern Europe ( GBIF Secretariat, 2022). Populations in the UK where it is also widespread ( NBN Atlas Partnership, 2021) appear to be on the decline ( Conrad *et al.*, 2006), with the conflicting trends of substantial increase in distribution towards to East, and a major decline in abundance since 1970 ( Randle *et al.*, 2019). 

*A. nigra* appears not to have been used in molecular phylogenies, and the genome will be useful to explore its evolution. The sister group of the genus *Aporophyla* Guenée, 1841 seems not to be established. It is currently placed in the tribe *Xylenini*.

The genome sequence should not only be useful in phylogeny but in studies of speciation. On BOLD there is a single quite variable (with up to about 1.28% divergence with the BIN) DNA barcode cluster, BOLD:AAC6249 (15 March 2023). This is reciprocally about 3.37% pairwise divergent from that of the Feathered Brindle, *A. australis* (BOLD:AAF5626), and then about 5.2% pairwise divergent to the pair *A. lutulenta* ([Denis & Schiffermüller], 1775) and *A. lueneburgensis* (Freyer, 1848). A genome assembly is also available for the latter (GCA_932294355.1) ( Boyes & Holland, 2023).

The genome of *Aporophyla nigra* was sequenced as part of the Darwin Tree of Life Project, a collaborative effort to sequence all named eukaryotic species in the Atlantic Archipelago of Britain and Ireland. Here we present a chromosomally complete genome sequence for *Aporophyla nigra*, based on one male specimen from Beinn Eighe National Nature Reserve, Scotland, UK.

## Genome sequence report

**Figure 1.**
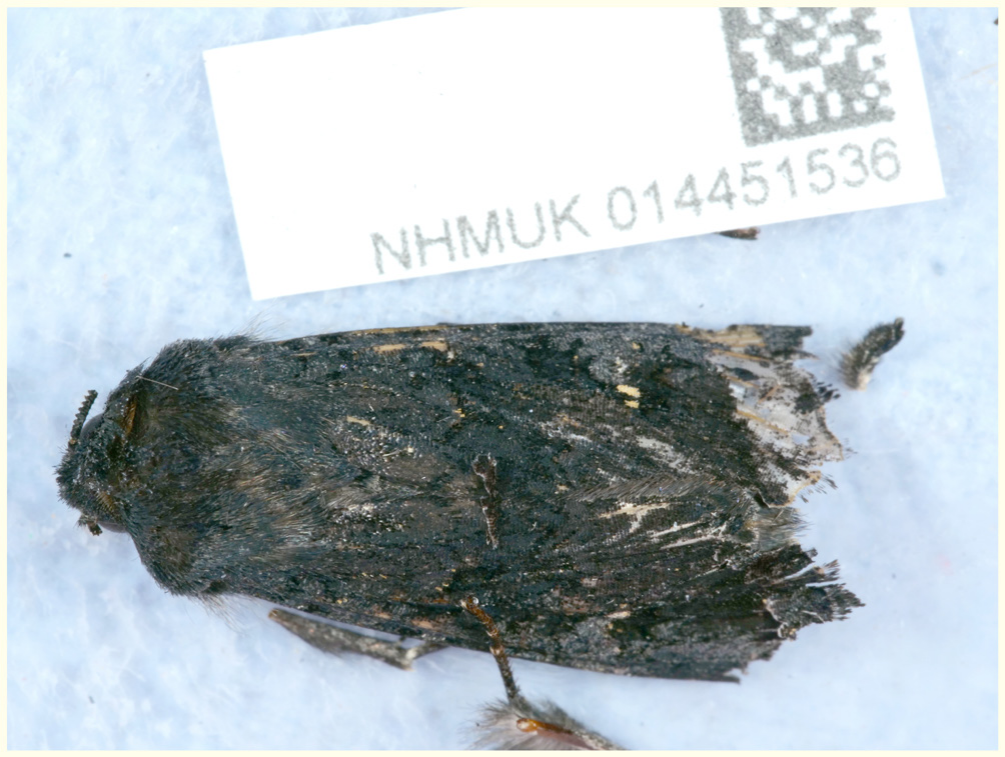
Photograph of the *Aporophyla nigra* (ilApoNigr3) specimen used for genome sequencing.

The genome was sequenced from one male *Aporophyla nigra* specimen ( Figure 1) collected from Beinn Eighe National Nature Reserve, Scotland, UK (latitude 57.63, longitude –5.35). A total of 28-fold coverage in Pacific Biosciences single-molecule HiFi long reads was generated. Primary assembly contigs were scaffolded with chromosome conformation Hi-C data. Manual assembly curation corrected 10 missing or mis-joins and removed six haplotypic duplications, reducing the scaffold number by 4.55%.

**Figure 2.**
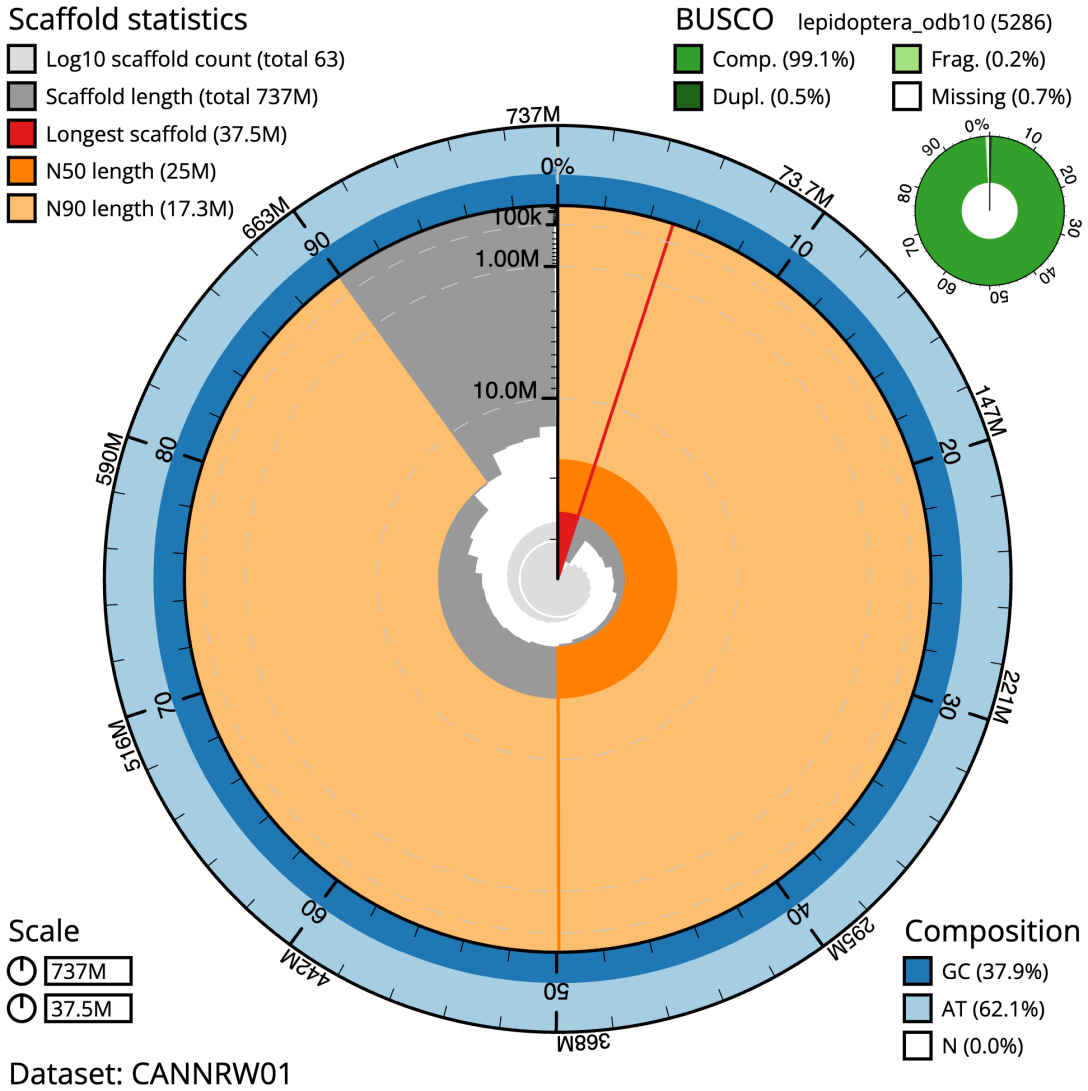
Genome assembly of *Aporophyla nigra*, ilApoNigr3.1: metrics. The BlobToolKit Snailplot shows N50 metrics and BUSCO gene completeness. The main plot is divided into 1,000 size-ordered bins around the circumference with each bin representing 0.1% of the 736,890,111 bp assembly. The distribution of scaffold lengths is shown in dark grey with the plot radius scaled to the longest scaffold present in the assembly (37,503,119 bp, shown in red). Orange and pale-orange arcs show the N50 and N90 scaffold lengths (24,958,797 and 17,300,924 bp), respectively. The pale grey spiral shows the cumulative scaffold count on a log scale with white scale lines showing successive orders of magnitude. The blue and pale-blue area around the outside of the plot shows the distribution of GC, AT and N percentages in the same bins as the inner plot. A summary of complete, fragmented, duplicated and missing BUSCO genes in the lepidoptera_odb10 set is shown in the top right. An interactive version of this figure is available at https://blobtoolkit.genomehubs.org/view/Aporophyla%20nigra/dataset/CANNRW01/snail.

The final assembly has a total length of 736.9 Mb in 63 sequence scaffolds with a scaffold N50 of 25.0 Mb ( Table 1). Most (99.7%) of the assembly sequence was assigned to 31 chromosomal-level scaffolds, representing 30 autosomes, and the Z sex chromosome. Chromosome-scale scaffolds confirmed by the Hi-C data are named in order of size ( Figure 2– Figure 5; Table 2). While not fully phased, the assembly deposited is of one haplotype. Contigs corresponding to the second haplotype have also been deposited.

**Table 1.** Genome data for *Aporophyla nigra*, ilApoNigr3.1.

Project accession data		
Assembly identifier	ilApoNigr3.1	
Species	*Aporophyla nigra*	
Specimen	ilApoNigr3	
NCBI taxonomy ID	988065	
BioProject	PRJEB56487	
BioSample ID	SAMEA110038400	
Isolate information	ilApoNigr3, male: whole organism (genome sequencing, Hi-C scaffolding and RNA sequencing)	
Assembly metrics ^*^		*Benchmark*
Consensus quality (QV)	66.2	*≥ 50*
*k*-mer completeness	100%	*≥ 95%*
BUSCO ^**^	C:99.1%[S:98.5%,D:0.5%], F:0.2%,M:0.7%,n:5,286	*C ≥ 95%*
Percentage of assembly mapped to chromosomes	99.7%	*≥ 95%*
Sex chromosomes	Z chromosome	*localised homologous pairs*
Organelles	Mitochondrial genome assembled	*complete single alleles*
Raw data accessions		
PacificBiosciences SEQUEL II	ERR10357395	
Hi-C Illumina	ERR10323138	
PolyA RNA-Seq Illumina	ERR10908611	
Genome assembly		
Assembly accession	GCA_947507805.1	
*Accession of alternate haplotype*	GCA_947458395.1	
Span (Mb)	736.9	
Number of contigs	121	
Contig N50 length (Mb)	11.9	
Number of scaffolds	63	
Scaffold N50 length (Mb)	25.0	
Longest scaffold (Mb)	37.5	

* Assembly metric benchmarks are adapted from column VGP-2020 of “Table 1: Proposed standards and metrics for defining genome assembly quality” from ( Rhie *et al.*, 2021). ** BUSCO scores based on the lepidoptera_odb10 BUSCO set using v5.3.2. C = complete [S = single copy, D = duplicated], F = fragmented, M = missing, n = number of orthologues in comparison. A full set of BUSCO scores is available at https://blobtoolkit.genomehubs.org/view/ilApoNigr3.1/dataset/CANNRW01/busco.

**Figure 3.**
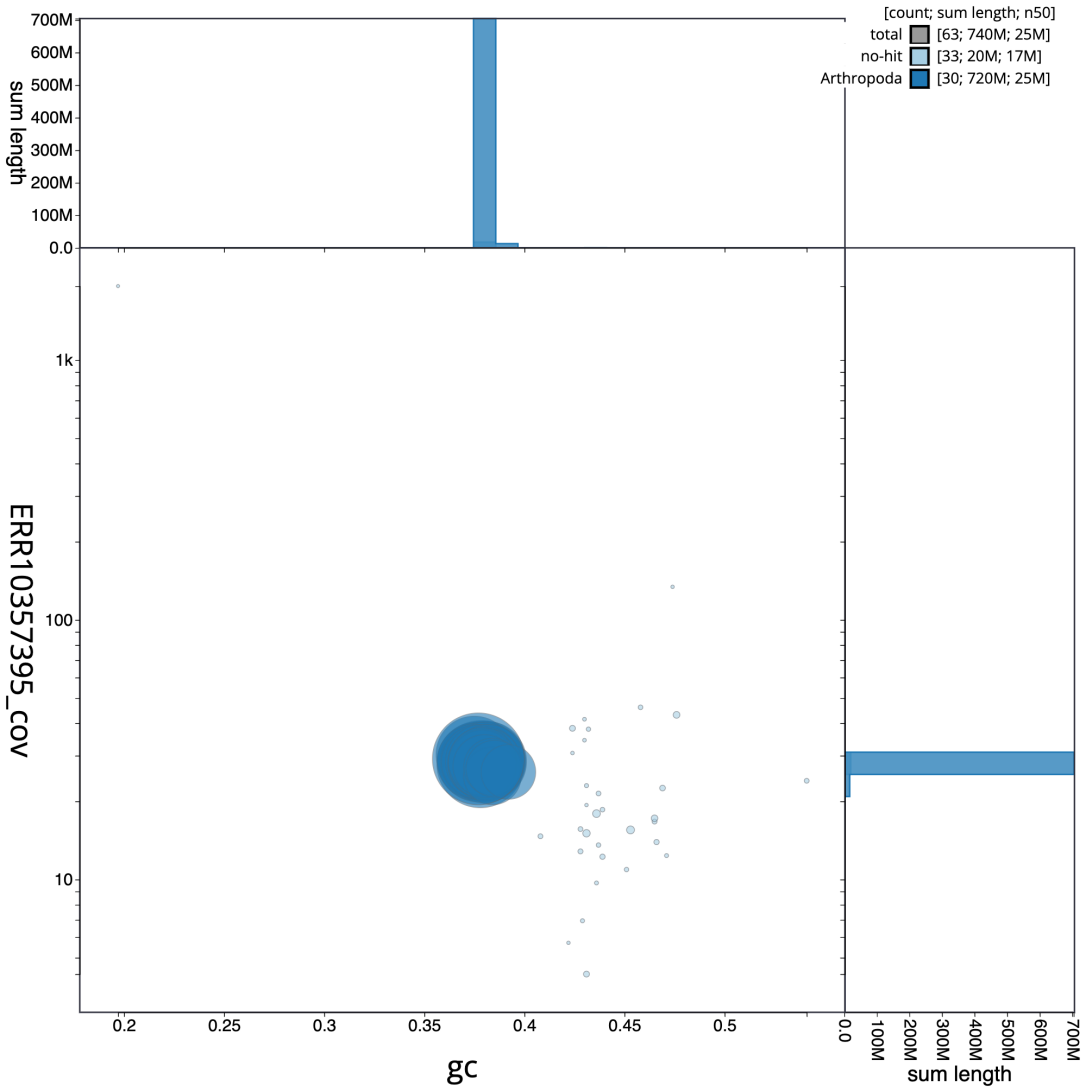
Genome assembly of *Aporophyla nigra*, ilApoNigr3.1: BlobToolKit GC-coverage plot. Scaffolds are coloured by phylum. Circles are sized in proportion to scaffold length. Histograms show the distribution of scaffold length sum along each axis. An interactive version of this figure is available at https://blobtoolkit.genomehubs.org/view/Aporophyla%20nigra/dataset/CANNRW01/blob.

**Figure 4.**
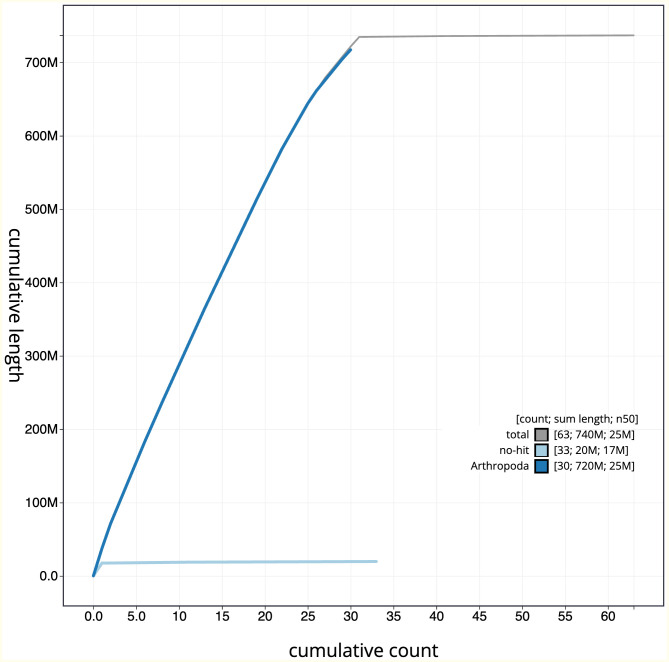
Genome assembly of *Aporophyla nigra*, ilApoNigr3.1: BlobToolKit cumulative sequence plot. The grey line shows cumulative length for all scaffolds. Coloured lines show cumulative lengths of scaffolds assigned to each phylum using the buscogenes taxrule. An interactive version of this figure is available at https://blobtoolkit.genomehubs.org/view/Aporophyla%20nigra/dataset/CANNRW01/cumulative.

**Figure 5.**
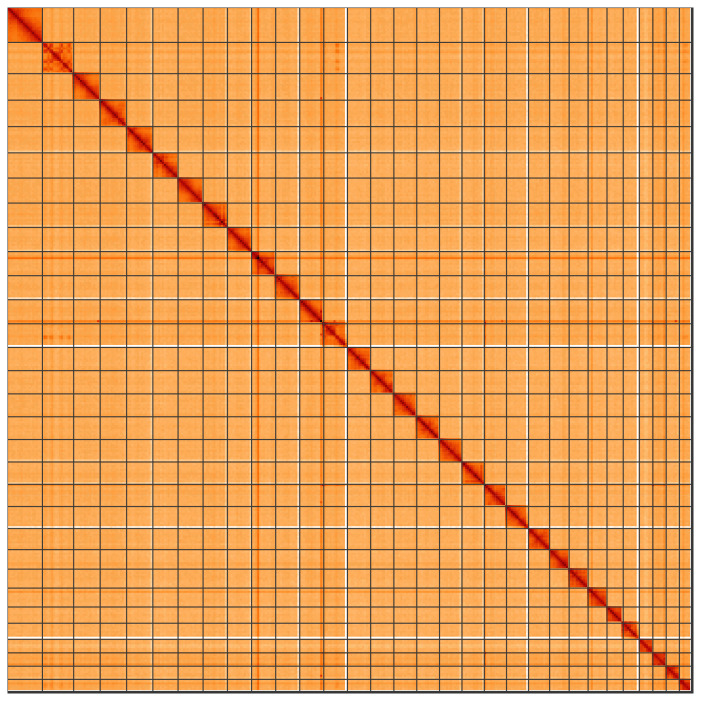
Genome assembly of *Aporophyla nigra*, ilApoNigr3.1: Hi-C contact map of the ilApoNigr3.1 assembly, visualised using HiGlass. Chromosomes are shown in order of size from left to right and top to bottom. An interactive version of this figure may be viewed at https://genome-note-higlass.tol.sanger.ac.uk/l/?d=WB-n_kKRQb6cpEmMMIF1DA.

**Table 2.** Chromosomal pseudomolecules in the genome assembly of *Aporophyla nigra*, ilApoNigr3.

INSDC accession	Chromosome	Size (Mb)	GC%
OX382292.1	1	33.6	37.8
OX382293.1	2	28.46	38
OX382294.1	3	28.42	38.1
OX382295.1	4	28.16	37.7
OX382296.1	5	27.09	37.6
OX382297.1	6	26.88	38
OX382298.1	7	26.11	38
OX382299.1	8	25.99	37.7
OX382300.1	9	25.91	37.5
OX382301.1	10	25.83	37.7
OX382302.1	11	25.82	38
OX382303.1	12	25.39	38
OX382304.1	13	24.96	37.7
OX382305.1	14	24.95	37.8
OX382306.1	15	24.57	38.2
OX382307.1	16	24.44	37.5
OX382308.1	17	24.12	37.7
OX382309.1	18	24.02	37.9
OX382310.1	19	23.87	38.2
OX382311.1	20	23.66	38
OX382312.1	21	22.6	38.1
OX382313.1	22	20.97	37.9
OX382314.1	23	20.36	37.9
OX382315.1	24	20.31	37.9
OX382316.1	25	17.43	38.2
OX382317.1	26	17.3	38
OX382318.1	27	14.5	38.4
OX382319.1	28	14.35	38.4
OX382320.1	29	14.14	38.5
OX382321.1	30	12.99	39.2
OX382291.1	Z	37.5	37.7
OX382322.1	MT	0.02	19.7

The estimated Quality Value (QV) of the final assembly is 66.2 with *k*-mer completeness of 100%, and the assembly has a BUSCO v5.3.2 completeness of 99.1% (single = 98.5%, duplicated = 0.5%), using the lepidoptera_odb10 reference set ( *n* = 5,286).

Metadata for specimens, spectral estimates, sequencing runs, contaminants and pre-curation assembly statistics can be found at https://links.tol.sanger.ac.uk/species/988065.

## Methods

### Sample acquisition and nucleic acid extraction

A male *Aporophyla nigra* specimen (specimen number NHMUK014451536, ToLID ilApoNigr3) was collected from Beinn Eighe National Nature Reserve, Scotland, UK (latitude 57.63, longitude –5.35) on 9 September 2021. The specimen was collected by David Lees (Natural History Museum) using a light trap. The specimen was identified by the collector and snap-frozen at –80°C.

DNA was extracted at the Tree of Life laboratory, Wellcome Sanger Institute (WSI). The ilApoNigr3 sample was weighed and dissected on dry ice with tissue set aside for Hi-C and RNA sequencing. Whole organism tissue was cryogenically disrupted to a fine powder using a Covaris cryoPREP Automated Dry Pulveriser, receiving multiple impacts. High molecular weight (HMW) DNA was extracted using the Qiagen MagAttract HMW DNA extraction kit. HMW DNA was sheared into an average fragment size of 12–20 kb in a Megaruptor 3 system with speed setting 30. Sheared DNA was purified by solid-phase reversible immobilisation using AMPure PB beads with a 1.8X ratio of beads to sample to remove the shorter fragments and concentrate the DNA sample. The concentration of the sheared and purified DNA was assessed using a Nanodrop spectrophotometer and Qubit Fluorometer and Qubit dsDNA High Sensitivity Assay kit. Fragment size distribution was evaluated by running the sample on the FemtoPulse system.

RNA was extracted from tissue of ilApoNigr3 in the Tree of Life Laboratory at the WSI using TRIzol, according to the manufacturer’s instructions. RNA was then eluted in 50 μl RNAse-free water and its concentration was assessed using a Nanodrop spectrophotometer and Qubit Fluorometer using the Qubit RNA Broad-Range (BR) Assay kit. Analysis of the integrity of the RNA was done using Agilent RNA 6000 Pico Kit and Eukaryotic Total RNA assay.

### Sequencing

Pacific Biosciences HiFi circular consensus libraries were constructed according to the manufacturers’ instructions. Poly(A) RNA-Seq libraries were constructed using the NEB Ultra II RNA Library Prep kit. DNA and RNA sequencing was performed by the Scientific Operations core at the WSI on Pacific Biosciences SEQUEL II (HiFi) and Illumina NovaSeq 6000 (RNA-Seq) instruments. Hi-C data were also generated from tissue of ilApoNigr3 using the Arima v2 kit and sequenced on the Illumina NovaSeq 6000 instrument.

### Genome assembly, curation and evaluation

Assembly was carried out with Hifiasm ( Cheng *et al.*, 2021) and haplotypic duplication was identified and removed with purge_dups ( Guan *et al.*, 2020). The assembly was scaffolded with Hi-C data ( Rao *et al.*, 2014) using YaHS ( Zhou *et al.*, 2023). The assembly was checked for contamination as described previously ( Howe *et al.*, 2021). Manual curation was performed using HiGlass ( Kerpedjiev *et al.*, 2018) and Pretext ( Harry, 2022). The mitochondrial genome was assembled using MitoHiFi ( Uliano-Silva *et al.*, 2022), which performed annotation using MitoFinder ( Allio *et al.*, 2020). To evaluate the assembly, MerquryFK was used to estimate consensus quality (QV) scores and *k*-mer completeness ( Rhie *et al.*, 2020). The genome was analysed and BUSCO scores ( Manni *et al.*, 2021; Simão *et al.*, 2015) were calculated within the BlobToolKit environment ( Challis *et al.*, 2020). Table 3 contains a list of software tool versions and sources.

**Table 3.** Software tools: versions and sources.

Software tool	Version	Source
BlobToolKit	4.0.7	https://github.com/ blobtoolkit/blobtoolkit
BUSCO	5.3.2	https://gitlab.com/ezlab/ busco
Hifiasm	0.16.1-r375	https://github.com/ chhylp123/hifiasm
HiGlass	1.11.6	https://github.com/ higlass/higlass
Merqury	MerquryFK	https://github.com/ thegenemyers/MERQURY. FK
MitoHiFi	2	https://github.com/ marcelauliano/MitoHiFi
PretextView	0.2	https://github.com/wtsi- hpag/PretextView
purge_dups	1.2.3	https://github.com/ dfguan/purge_dups
YaHS	yahs-1.1.91eebc2	https://github.com/ c-zhou/yahs

### Ethics and compliance issues

The materials that have contributed to this genome note have been supplied by a Darwin Tree of Life Partner. The submission of materials by a Darwin Tree of Life Partner is subject to the Darwin Tree of Life Project Sampling Code of Practice. By agreeing with and signing up to the Sampling Code of Practice, the Darwin Tree of Life Partner agrees they will meet the legal and ethical requirements and standards set out within this document in respect of all samples acquired for, and supplied to, the Darwin Tree of Life Project. All efforts are undertaken to minimise the suffering of animals used for sequencing. Each transfer of samples is further undertaken according to a Research Collaboration Agreement or Material Transfer Agreement entered into by the Darwin Tree of Life Partner, Genome Research Limited (operating as the Wellcome Sanger Institute), and in some circumstances other Darwin Tree of Life collaborators.

## Data Availability

European Nucleotide Archive: *Aporophyla nigra*. Accession number PRJEB56487; https://identifiers.org/ena.embl/PRJEB56487. ( Wellcome Sanger Institute, 2022) The genome sequence is released openly for reuse. The *Aporophyla nigra* genome sequencing initiative is part of the Darwin Tree of Life (DToL) project. All raw sequence data and the assembly have been deposited in INSDC databases. The genome will be annotated using available RNA-Seq data and presented through the Ensembl pipeline at the European Bioinformatics Institute. Raw data and assembly accession identifiers are reported in Table 1.

## Author information

Members of the Natural History Museum Genome Acquisition Lab are listed here: https://doi.org/10.5281/zenodo.4790042.

Members of the Darwin Tree of Life Barcoding collective are listed here: https://doi.org/10.5281/zenodo.4893703.

Members of the Wellcome Sanger Institute Tree of Life programme are listed here: https://doi.org/10.5281/zenodo.4783585.

Members of Wellcome Sanger Institute Scientific Operations: DNA Pipelines collective are listed here: https://doi.org/10.5281/zenodo.4790455.

Members of the Tree of Life Core Informatics collective are listed here: https://doi.org/10.5281/zenodo.5013541.

Members of the Darwin Tree of Life Consortium are listed here: https://doi.org/10.5281/zenodo.4783558.
