# Supplementary material for: The genome sequence of the Black Rustic, Aporophyla nigra (Haworth, 1809)
Source: Wellcome Open Res. 2023 Apr 17;8:173. [Version 1] doi: 10.12688/wellcomeopenres.19339.1 (PMC12476537; doi:10.12688/wellcomeopenres.19339.1)
Supplement: Peer review history

# Reviewer report: Reviewer response for version 1

**DOI:** 10.21956/wellcomeopenres.21424.r134299

**Published:** 2025-09-27

**Authors:** Deniz Aygören Uluer

**Affiliations:** 1 Ahi Evran University, Kirşehir, Turkey

Dear authors,

 Here are a few problems to revise:

 1-The Black Rustic, Aporophyla nigra, is a medium sized…

 Author of the species is missing

 2-A. nigra is found in a wide variety of open habitats including those in woodlands. The larva is polyphagous on a wide range of herbaceous plants and shrubs. Adults seek nectar such as that of Ivy (Hedera helix L.) at night, and also feed on ripe blackberries.

 Citations are missing

 3- It is currently placed in the tribe Xylenini.

 Citation is missing

 4-The genome was sequenced from one male Aporophyla nigra specimen (Figure 1) collected from Beinn Eighe National Nature Reserve, Scotland, UK (latitude 57.63, longitude –5.35).

 Did you prepare a voucher specimen? If so, where is it deposited?

 5- Figure 1. Photograph of the Aporophyla nigra (ilApoNigr3) specimen used for genome sequencing.

 Aporophyla nigra should be italics

Are sufficient details of methods and materials provided to allow replication by others?

Yes

Is the rationale for creating the dataset(s) clearly described?

Yes

Are the datasets clearly presented in a useable and accessible format?

Yes

Are the protocols appropriate and is the work technically sound?

Yes

Reviewer Expertise:

Plant phylogenetics

I confirm that I have read this submission and believe that I have an appropriate level of expertise to confirm that it is of an acceptable scientific standard.

# Reviewer report: Reviewer response for version 1

**DOI:** 10.21956/wellcomeopenres.21424.r61447

**Published:** 2023-07-05

**Authors:** Jerome H Hui

**Affiliations:** 1 Chinese University of Hong Kong, Hong Kong, China

Lees and colleagues report the genome sequence of black rustic *Aporophyla nigra*. This species can be commonly found in Britain. Molecular data of this species are scarce prior to this report, and are mainly confined to COI sequences deposited to the NCBI database. This new genome resource will be very useful for further studies, such as understanding their evolutionary relationships with other moths.

 This genome resource is excellent from the summary statistics, with high BUSCO numbers, high sequence continuity (scaffold N50), and majority of sequences contained on the 31 pseudochromosomes (plus mitochondrion). To sum up, this is a valuable contribution.

Are sufficient details of methods and materials provided to allow replication by others?

Yes

Is the rationale for creating the dataset(s) clearly described?

Yes

Are the datasets clearly presented in a useable and accessible format?

Yes

Are the protocols appropriate and is the work technically sound?

Yes

Reviewer Expertise:

Genomics, evolution, invertebrates

I confirm that I have read this submission and believe that I have an appropriate level of expertise to confirm that it is of an acceptable scientific standard.
